# Comparing three short questionnaires to detect psychosocial problems among 3 to 4-year olds

**DOI:** 10.1186/s12887-015-0391-y

**Published:** 2015-07-16

**Authors:** Meinou H.C. Theunissen, Anton G.C. Vogels, Marianne S. de Wolff, Mathilde R. Crone, Sijmen A. Reijneveld

**Affiliations:** TNO Child Health, P. O. Box 2215, 2301 CE, Leiden, NL The Netherlands; Department of Public Health and Primary Care, Leiden University Medical Center (LUMC), Leiden, The Netherlands; Department of Health Sciences, University Medical Center Groningen, University of Groningen, Groningen, NL The Netherlands

## Abstract

**Background:**

Validated questionnaires help community pediatric services to identify psychosocial problems. Our aim was to assess which of three short questionnaires was most suitable for this identification among pre-school children.

**Methods:**

We included 1,650 children (response 64 %) aged 3–4 years undergoing routine well-child health assessments in 18 services across the Netherlands. Child healthcare professionals (CHPs) interviewed and examined children and parents. Parents were randomized regarding filling out the Strengths and Difficulties Questionnaire (SDQ) or the KIPPPI, a Dutch-origin questionnaire. In addition, all filled out the Ages and Stages Questionnaires: Social-Emotional (ASQ:SE) and the Child Behavior Checklist (CBCL). We assessed the internal consistency and validity of each questionnaire, with CBCL and treatment status as criteria, and the degree to which each questionnaire could improve identification based solely on clinical assessment.

**Results:**

The internal consistency of the total problems scale of each questionnaire was satisfactory, Cronbach's alphas varied between 0.75 and 0.98. Only the SDQ discriminated sufficiently between children with and without problems as measured by the CBCL (sensitivity = 0.76 at a cut-off point with specificity = 0.90), in contrast to the other two questionnaires (with sensitivity indices varying between 0.51–0.63). Similar results were found for the treatment status criterion, although sensitivity was lower for all questionnaires. The SDQ seemed to add most to the identification of psychosocial problems by CHPs, but the differences between the SDQ and the ASQ:SE were not statistically significant.

**Conclusions:**

The SDQ is the best tool for the identification of psychosocial problems in pre-school children by community paediatric services.

## Background

Around 10 to 25 % of children have emotional or behavioural problems [1, 2]. Recent studies have shown that only one in five of the identified children with emotional or behavioural problems receive psychosocial care [1, 3]. Children with psychosocial problems are likely to experience difficulties in various aspects of their daily functioning. These problems may be severe and persist over time [4]. Early detection and treatment can improve the prognosis for psychosocial problems in children [5, 6].

Community paediatric services are important for the early identification of psychosocial problems in children because they offer routine healthcare services to the population as a whole. In the Netherlands, physicians and nurses (Child Healthcare Professionals, CHPs) working in the Preventive Child Healthcare system (PCH) routinely offer preventive healthcare to all children aged 0–19, similar to well-child care in the United States. The PCH system is important for the early identification of psychosocial problems. It was shown, however, that CHPs failed to identify psychosocial problems in about half the children with parent-reported problems on the Child Behaviour Checklist (CBCL) questionnaire, when they had to base their judgment solely on a clinical assessment [1, 7].

Validated questionnaires may improve the identification of psychosocial problems by CHPs [8]. An example of such a questionnaire is the CBCL, a highly reliable and valid instrument for assessing psychosocial problems in children [9–11]. However, the CBCL questionnaire is too long to be used as a routine screening questionnaire in community paediatric services. Daily practice in these services requires short instruments.

Several short parent-reported questionnaires are available for use among children three and four years old: the Ages and Stages Questionnaires: Social-Emotional (ASQ:SE) [12], the Strengths and Difficulties Questionnaire 3–4 (SDQ 3–4) [13, 14], and the KIPPPI 1–4 [15]. Evidence on the validity and reliability of these questionnaires lacks, though this is necessary for adequate preventive services. First, missing children with psychosocial problems causes them to lack potentially beneficial early treatment. Second, labelling of children as having psychosocial problems when they do not have them may cause unwanted side-effects such as stigma and the useless provision of care.

The ASQ:SE questionnaires span the 3 to 66-month period with 8 separate assessment intervals. It is a promising questionnaire because the psychometric properties of the ASQ:SE have been shown to be good in the USA [16]. However, the Dutch version has not yet been investigated. The psychometric properties of the SDQ Parent Form PF have shown to be good for children 3 and 4 years old in the Netherlands [17]. These properties has been shown to be good for older children (4–16 years) as well, in many countries [8, 13, 14, 18–25]. No published studies are available that investigate the psychometric properties of the KIPPPI 1–4 (a Dutch questionnaire, the acronym standing for ‘short instrument for the psychological and pedagogical inventory’). The validity results of the KIPPPI-version for younger children (toddler KIPPPI) were inconsistent. Wolff et al. found for the toddler KIPPPI in two years olds lower validity indices than Kruizinga et al. found [26, 27].

The aim of this study was to assess which of three short questionnaires (SDQ, KIPPPI, ASQ:SE) was most suitable for the identification of psychosocial problems among 3–4 years old children. We compare the psychometric properties (internal consistency, scale structure and validity) and the added value of these questionnaires regarding routine identification by PCH. The questionnaires were validated using the following criteria: an elevated CBCL score and current receiving treatment for psychosocial problems.

## Methods

### Population

The sample was obtained in a two-stage procedure. In the first step, all PCH services in the Netherlands were asked to participate in this study (at that time 55); 18 agreed to do so. The participating PCH services were located throughout the country. In the second step, each of the participating PCH services was required to provide a random sample of children aged 36 and 45 months who were invited for a routine well-child examination.

A total of 2575 parents were asked to participate in this study: 17.2 % explicitly refused to participate, 15.7 % did not return the questionnaire and 3.0 % did not provide complete data on the questionnaires, resulting in a response of 1650 parents who provided data for their child (64.1 %). Respondents were representative of the total sample in terms of gender and family composition, but non-response was higher for children of immigrant origin (compared with children from Dutch origin) and for children four years of age (compared with children three years of age). According to the Cohen effect size index *w* differences between responders and non-responders with regard to child ethnicity, age, gender and family composition were small with *w* varying between 0.02–0.16.

The study was approved by the Medical Ethics Committee of Leiden University Medical Center (LUMC).

### Randomization

Parents were randomized as to whether they filled out the SDQ or the KIPPPI. To randomize, we used a previously developed procedure [8]. In that way, we obtained complete equivalence of data. Randomization was necessary because otherwise the set of questionnaires would become too long. Therefore, the SDQ or KIPPPI was combined with the ASQ:SE, the shortest of the three questionnaires and the one with the largest age range (3 to 66-month period) among pre-school children. Randomization led to two subsamples, the first one completed the CBCL, ASQ:SE and the SDQ, the second one completed the CBCL, ASQ:SE and the KIPPPI.

### Procedure and measurements

The data were collected during the routine well-child examinations, between August 2008 and June 2011. The questionnaires were mailed to parents along with the standard invitation for the preventive health assessment. They were filled in at home. The completed questionnaires were returned to the CHP in a sealed envelope and forwarded to the research institute without being opened.

The CHP then took a routine history and physically assessed each child. In order to measure clinical assessment and treatment status the CHP answered the following questions after each assessment: ‘Does the child have a psychosocial problem at this moment?’ (yes or no) and ‘Does the child currently receive treatment for psychosocial problems?’. The first question was used to assess the degree to which each instrument can improve the identification of children with problems based solely on clinical assessment by the CHP. The last question was used a measure of ‘treatment status’, one of the two criterion measures. The CHP also provided data about child age and gender, ethnic background, family composition, parental employment and educational level, number of siblings, and maternal and paternal age. Parental educational level was the highest level of education completed successfully by a parent. Family composition concerned the number of parents in the family (two parents or one parent). These background characteristics are presented in Table 1.

**Table 1:** Demographic characteristics of the participating children

	SDQ (*n* = 839)	KIPPPI (*n* = 832)	ASQ:SE (*n* = 1650)##
	*n* (%)	*n* (%)	*n* (%)
Background characteristics#			
Gender			
Boy	428 (51.0 %)	431 (51.9 %)	851 (51.6 %)
Girl	411 (49.0 %)	400 (48.1 %)	798 (48.4 %)
Child’s age			
3 years	506 (61.2 %)	465 (56.2 %)	959 (58.7 %)
4 years	321 (38.8 %)	362 (43.8 %)	675 (41.3 %)
Ethnicity			
Dutch-born	692 (85.4 %)	705 (88.2 %)	1379 (86.8 %)
Immigrant	118 (14.6 %)	94 (11.9 %)	210 (13.2 %)
Family composition			
Two parents	766 (96.0 %)	738 (94.7 %)	1484 (95.3 %)
One parent	32 (4.0 %)	41 (5.3 %)	73 (4.7 %)
Employment status			
Unemployed	10 (1.3 %)	15 (2.0 %)	25 (1.7 %)
One full-time job	165 (21.3 %)	153 (20.4 %)	315 (20.9 %)
One full-time + one part-time job	505 (65.2 %)	496 (66.1 %)	986 (65.5 %)
Both part-time	65 (8.4 %)	65 (8.7 %)	128 (8.5 %)
Both full-time	30 (3.9 %)	21 (2.8 %)	51 (3.4 %)
Parental educational level			
Lower education	100 (12.3 %)	88 (11.0 %)	186 (11.7 %)
Medium education	321 (39.6 %)	308 (38.6 %)	619 (39.0 %)
Higher education	389 (48.0 %)	401 (50.3 %)	783 (49.3 %)
Number of siblings			
No sibs	206 (24.6 %)	217 (26.1 %)	417 (25.3 %)
1 sib	441 (52.6 %)	415 (49.9 %)	850 (51.5 %)
2 and > sibs	192 (22.9 %)	200 (24.0 %)	383 (23.2 %)
Maternal age at birth			
19–30	176 (21.1 %)	177 (21.4 %)	350 (21.3 %)
31–35	336 (40.3 %)	309 (37.4 %)	641 (39.1 %)
36–40	257 (30.8 %)	281 (34.0 %)	527 (32.1 %)
>40	65 (7.8 %)	60 (7.3 %)	122 (7.4 %)
Paternal age at birth			
22–30	83 (10.1 %)	78 (9.6 %)	159 (9.9 %)
31–35	241 (29.5 %)	219 (26.9 %)	455 (28.2 %)
36–40	325 (39.7 %)	344 (42.3 %)	661 (41.0 %)
>40	169 (20.7 %)	172 (21.2 %)	336 (20.9 %)
Elevated CBCL score	84 (10.0 %)	80 (9.6 %)	163 (9.9 %)

# Number of omissions: Gender (*n* = 1), Child’s age (*n* = 16), Ethnicity (*n* = 61), Family composition (*n* = 93), Employment status (*n* = 145), Parental educational level (*n* = 62), Number of siblings (*n* = 0), Maternal age (*n* = 10), Paternal age (*n* = 40)

## The total number of children does not add up to *n* = 1,671, because of omissions on the ASQ:SE questionnaire

Currently receiving treatment for psychosocial problems and an elevated CBCL (1.5-5) Total Problems Score (TPS) were used as the criteria for the occurrence of psychosocial problems. Currently receiving treatment was used as a criterion variable because this study did not include a clinical assessment criterion. Clinical assessments are expensive and time-consuming and therefore not appropriate for research comprising large populations. The CBCL assesses parental reports about children’s behavioural and emotional problems in the preceding two months. Its reliability and validity have been found to be good, also in the Netherlands [9–11]. The CBCL comprises 99 problem items that are used to compute Total, Internalizing and Externalizing problem scores. Children were allocated to a normal range or an elevated range using the 90th percentile cut-off point in this sample.

The SDQ PF 3–4 was developed in Great Britain [13, 14, 24, 25]. It consists of 25 items relating to children’s strengths and difficulties. Each item has to be scored on a 3-point scale (0 = ‘not true’, 1 = ‘somewhat true’ and 2 = ‘certainly true’). Items can be grouped in five subscales: emotional symptoms, conduct problems, hyperactivity-inattention, peer problems, and pro-social behaviour. A SDQ Total Difficulties Score (TDS) can be calculated by adding up the scores for the first four sub-scales.

The ASQ:SE was developed in the USA as a complement to the Ages and Stages Questionnaires (ASQ), a general developmental screening tool for children [28]. The ASQ:SE addresses the social and emotional behaviour of children ranging in age from 3 to 66 months. We used the 36 months (31 items) and 48 months (33 item) version [12]. Each item has to be scored on a 3-point scale (0 = ‘never or rarely’, 5 = ‘sometimes’ and 10 = ‘most of the time’). An additional 5 points are given for items where parents indicate that the behavior is of concern to them. Scores for each item are then combined into a total score. No official Dutch version of the ASQ:SE was available. Therefore, this questionnaire was translated following a procedure advised by Guillemin, using three native language translators and –independent- back-translators [29]. Translations were then compared on the basis of the back translations, leading to the selection of the best translation in Dutch.

The KIPPPI 1–4 is a Dutch instrument for parents, designed specifically for Dutch PCH. It contains 70 items, relating to children’s social emotional development, well-being and behaviour. Each item has to be scored on a 4-point scale (1 = ‘almost never’, 2 = ‘sometimes’ 3 = ‘often’ and 4 = ‘almost always’). These items allow for the calculation of three subscales (competence, autonomy, well-being) and a total difficulties scale (TDS).

### Analysis

The analyses included the assessment of the internal consistency, scale structure, the validity and the added value of the instruments for the identification of psychosocial problems. We first computed the internal consistency (Cronbach’s alpha). Next, we examined the fit between the scale structure and the observed data with confirmatory factor analyses (CFA) using Mplus Structural Equation Modelling [30]. In the CFA, the models were considered as fitting when the Comparative Fit Index (CFI) was higher than 0.90. Because the CFI index is a strict criterion, we considered the model as an approximating fit when the Root Mean Square Error of Approximation (RMSEA) was less than 0.08.

The validity of the instruments was assessed with sensitivity and specificity indices, using CBCL TPS, and ‘current treatment for psychosocial problems’ as the criteria. No established cut-off points were available for the SDQ 3–4, KIPPPI 1–4 and the ASQ:SE. Therefore, first, we calculated the AUCs (Area Under receiver operation Curves) for each questionnaire which shows sensitivity and specificity for all scores, using the elevated CBCL TPS as criterion. Next, we chose an appropriate cut-off point, namely the score that was associated with a specificity of at least 0.90 in our sample, to determine sensitivity indices and related test characteristics. Third, the added value of the instruments was determined. We assessed the degree to which each instrument can improve the identification of children with problems based solely on clinical assessment by the CHP without knowledge of the instrument. Logistic regression analyses were performed for each instrument with the CBCL criterion measure as the dependent variable. In the first step the identification by a CHP was included in the analyses and in the second step the dichotomized score on the instrument was added as an independent variable. The criterion ‘current treatment for psychosocial problems’ was not used in the added value analyses, since psychosocial problems of children currently being treated were known to CHPs.

Finally, we repeated all logistic regression analyses excluding children currently receiving treatment for psychosocial problems, because the identification of children with problems is most relevant for those children who are not yet being treated for such problems.

## Results

### Background characteristics of the sample

The mean age of the sample was 40 months (standard deviation: 5 months). Further demographic information is presented in Table 1. We found no differences in background characteristics between the two subsamples (KIPPPI vs. SDQ) as indicated by Chi-square tests, except for child age. The KIPPPI sample comprised relatively more four-year-old children. According to the Cohen effect size index *w* differences between the two subsamples with regard to child age were small (*w* = 0.05).

### Internal consistency and scale structure

The Cronbach’s alphas of the total problem scales of the SDQ, KIPPPI and the ASQ:SE (36 and 45 months) were 0.78, 0.98, 0.77 and 0.75, respectively. The differences in Cronbach’s alphas could be explained by the numbers of items per questionnaire: corrected with the Spearman Brown Prophecy Formula Cronbach’s alphas were 0.93, 0.98, 0.88 and 0.86, for the SDQ, KIPPPI and the ASQ:SE (36 and 45 months), respectively. Cronbach’s alphas for the SDQ subscales varied between 0.50 and 0.74 and for the KIPPPI subscales between 0.36 and 0.99 (Table 2).

**Table 2:** Internal consistency of SDQ, KIPPPI and ASQ:SE total problems scale and subscales

	Number of items	Cronbach’s alpha
SDQ scales#		
Total difficulties	20	0.78
Emotional symptoms	5	0.54
Conduct problems	5	0.64
Hyperactivity	5	0.74
Peer problems	5	0.50
Prosocial	5	0.65
KIPPPI scales		
Total difficulties	70	0.98
Competence	28	0.86
Autonomy	10	0.36
Well-being	32	0.99
ASQ:SE		
Total 36 months	31	0.77
Total 45 months	33	0.75

#Previously reported in Theunissen et al. 2013 [17]

Structural equation modelling showed a poor fit of the single-scale models of the SDQ, the KIPPPI and the ASQ:SE according to the CFI criterion, but an approximating fit according to the RMSEA criterion (SDQ: CFI = 0.644, RMSEA = 0.079; KIPPPI: CFI = 0.352, RMSEA = 0.073; ASQ:SE 36: CFI = 0.603, RMSEA = 0.065; ASQ:SE 45: CFI = 0.600, RMSEA = 0.057). For the SDQ and the KIPPPI more subtle models were evaluated, reflecting the subscales of the questionnaires. For both questionnaires a poor fit was found according to the CFI criterion (SDQ = 0.682; KIPPPI = 0.456) and an approximate fit according to the RMSEA criterion (SDQ = 0.075; KIPPPI = 0.069), when using independent subscales assuming no correlation between the subscales.

### Validity

The cut-off points led to prevalence rates of elevated scores 13.8 % on the SDQ, 13.6 % on the KIPPPI, 15.3 % on the ASQ:SE 36 months, and 13.2 % on the ASQ:SE 48 months. Table 3 presents data on the validity of the three questionnaires, using an elevated CBCL and treatment status as criteria. The SDQ, KIPPPI and ASQ:SE scores correlated significantly with the CBCL scores. The highest correlation coefficient was found between the CBCL and the SDQ (Spearman’s r = 0.70) and the lowest one between the CBCL and the ASQ:SE (45 months) (Spearman’s r = 0.54).

**Table 3:** Test characteristics of the SDQ, KIPPPI and ASQ:SE using an elevated CBCL score and treatment status as criteria

	SDQ#	KIPPPI	ASQ:SE	
			36 months	45 months
Cut-off point	>10	>140	>52.5	>50.78
CBCL				
Spearman’s r	0.70	0.57	0.63	0.54
Kappa	0.59	0.35	0.48	0.43
Sensitivity (95 % CI)	0.76 (0.65–0.84)	0.51 (0.40–0.62)	0.65 (0.55–0.74)	0.63 (0.45–0.75)
Specificity (95 % CI)	0.93 (0.91–0.95)	0.90 (0.77–0.83)	0.91 (0.89–0.93)	0.91 (0.88–0.93)
AUC (95 % CI)	0.93 (0.90–0.96)	0.83 (0.78–0.87)	0.90 (0.88–0.93)	0.86 (0.81–0.92)
Treatment status				
Kappa	0.20	0.13	0.18	0.10
Sensitivity (95 % CI)	0.68 (0.46–0.85)	0.48 (0.28–0.68)	0.67 (0.47–0.83)	0.40 (0.20–0.63)
Specificity (95 % CI)	0.88 (0.86–0.90)	0.88 (0.86–0.90)	0.86 (0.84–0.88)	0.88 (0.85–0.90)
AUC (95 % CI)	0.90 (0.85–0.95)	0.76 (0.69–0.85)	0.84 (0.76–0.92)	0.78 (0.70–0.86)

Abbreviations: CI, Confidence Interval; AUC, Area Under receiver operation Curve; CBCL, Child Behavior Checklist

#Previously reported in Theunissen et al. 2013 [17]

Table 3 also presents the Cohen’s kappas, sensitivity and specificity indices for both criteria regarding the dichotomised total problems scores. Cohen’s kappas varied between 0.35 (KIPPPI) and 0.59 (SDQ) for the CBCL, and varied between 0.10 (ASQ:SE 45) and 0.20 (SDQ) for treatment status.

Due to the way we established the cut-off points the specificity of all scales for the CBCL criterion is about 0.90 or slightly higher. Sensitivity indices varied from 0.51 (KIPPPI) to 0.76 (SDQ) for the CBCL criterion. Sensitivity using treatment status as criterion, varied from 0.40 (ASQ:SE 45 months) to 0.68 (SDQ) using the same cut-offs. Specificity using treatment status as criterion varied between 0.86 and 0.88.

### Added value

Table 4 presents the findings regarding the added value of each questionnaire to the identification of the CHP. These show that an elevated score on each of the three questionnaires increased the likelihood that a child has an elevated CBCL score compared with only the clinical assessment by the CHP. The adjusted odds ratio (ORs) for all children were: SDQ 33.1 (18.1–60.8); KIPPPI 8.37 (4.99–14.1); ASQ:SE 36 months 15.5 (9.57–25.0); ASQ:SE 45 months 13.0 (6.85–24.5). Information on an elevated SDQ TDS offered most added value for the prediction of an elevated CBCL compared to the other questionnaires. However, the 95 % confidence intervals of the adjusted ORs of the SDQ and ASQ:SE overlapped with each other, indicating that there were no significant differences between these questionnaires. Repetition of these analyses for children not under treatment yielded similar results.

**Table 4:** Results from separate logistic regression analyses for each questionnaire on elevated CBCL TPS score, taking the identification by the CHP into account

	SDQ#	KIPPPI	ASQ:SE	
			36 months	45 months
	OR (95 % CI)	OR (95 % CI)	OR (95 % CI)	OR (95 % CI)
All children				
N	812	802	930	664
CHP detected problems yes (vs. no)	4.70 (2.46–8.98)	2.50 (1.41–4.44)	2.99 (1.75–5.11)	3.99 (2.04–7.80)
Elevated score on questionnaire yes (v. no)##	33.1 (18.0–60.8)	8.37 (4.99–14.1)	15.5 (9.57–25.0)	13.0 (6.85–24.5)
Children not receiving treatment				
N	787	777	900	644
CHP detected problems yes (vs. no)	4.45 (2.16–9.19)	1.98 (1.03–3.80)	2.19 (1.20–4.0)	4.12 (2.01–8.44)
Elevated score on questionnaire yes (vs. no)##	35.1 (18.6–66.3)	9.76 (5.64–16.9)	13.9 (8.45–22.9)	12.6 (6.47–24.3)

Adding elevated scores to the model always led to a significant change in the log likelihood ratio

#Previously reported in Theunissen et al. 2013 [17]

## Adjusted ORs, taking into account the identification of problems by the CHP

Abbreviation: *CBCL*, Child Behavior Checklist; *TPS*, Total Problem Score; *CHP*, Child Healthcare Professional; *OR*: odds ratio; *CI*: confidence interval

## Discussion

We compared the psychometric properties (internal consistency, scale structure and validity) of three questionnaires (SDQ, KIPPPI and ASQ:SE) and whether they could enhance the early detection of psychosocial problems among 3–4 years old children in community paediatric practice. Our findings showed that the internal consistencies of the total scales of all questionnaires were satisfactory. Regarding validity, only the SDQ discriminated sufficiently between 3–4 years old children with and without problems as measured by the CBCL, and not the other two questionnaires. Similar results were found for the treatment status criterion, although sensitivity was lower for all questionnaires using this criterion. The SDQ added more to the identification of psychosocial problems by CHPs than the KIPPPI and the ASQ:SE, although the differences between the SDQ and the ASQ:SE were not statistically significant.

### Fit with previous literature

In general, our findings on the internal consistency and validity of the SDQ were in line with those of Vogels et al. on the SDQ 4–16 in older children (ages 7–12 years) [8]. The internal consistencies (Cronbach’s alpha) of the total problem scales for both age versions was satisfactory: 0.78 for the SDQ 3–4 and 0.80 for the SDQ 4–16. The sensitivities and the AUCs (which measure the accuracy of the SDQ for the detection of problems) in both SDQ versions were in the same range. This implies that the validity of the SDQ was similar among pre-school children and school-age children.

Our results showed poorer psychometric properties of the ASQ:SE compared to findings in the USA. In the USA Cronbach’s alpha’s varied between 0.89–0.91, while in our study they varied between 0.75–0.77 on the 36 and 48 months versions [16]. Our results also showed a lower sensitivity of the ASQ:SE compared to findings in the USA. In the USA sensitivity varied between 0.77–0.89 at a specificity between 0.88–0.92 on the 36 and 48 months versions [16]. However, we found similar (36 months version) and even better psychometric properties (48 months version) of the ASQ:SE compared to findings on Korean children. In Korea sensitivity was 0.67 at a specificity of 0.96 on the 36 months version and 0.33 at a specificity of 0.93 on the 48 months version [31]. In our study the sensitivity varied between 0.63–0.65 at a specificity of 0.91 on the 36 and 48 months interval. What might explain the differences in psychometric properties between these countries? The ASQ:SE was developed in the USA, and in our study translated to Dutch. Cultural differences between the two countries and the translation of the ASQ:SE to Dutch might explain the differences in psychometric properties between the two countries. We previously showed, however, rather similar psychometric properties for Dutch version of the Pediatric Symptom Checklist (PSC), also developed in the US, among children aged 9–11 years [32]. Additional research is needed on the explanation of these differences.

We found a similar validity of the KIPPPI 1–4 as Wolff et al. found for the baby and toddler KIPPPI in younger children (ages 6, 14 and 24 months), [26] but lower validity indices than Kruizinga et al. found for the toddler KIPPPI [27]. In two years olds, they found a sensitivity of 0.74 at a specificity of 0.90. Additional research is needed to explain this heterogeneity.

We found rather low internal consistencies for some subscales of the SDQ and of the KIPPPI. Cronbach’s alphas varied between 0.48-0.73 for the SDQ 3–4 and between 0.36 and 0.99 for the KIPPPI. For the SDQ the low internal consistencies may be partly due to the small number of items (5) in each scale. Furthermore, the analyses investigating the scale structures of the questionnaires showed mediocre and negative Structural Equation Modelling results. This indicates that the items provide information that is not expressed in the subscale scores. More research is needed to investigate the subscale structure of the SDQ.

Only for the SDQ we found previous studies that assessed the added value, being (65.4, CI 24.8–172.4) for the SDQ 4–16 compared to 33.1 (CI 18.0–60.8) for the SDQ 3–4 [8]. The 95 %-CI overlap, i.e., these differences were not statistically significant. This implies that the added value of the SDQ to the identification of psychosocial problems by CHPs was similar among pre-school children and school-age children.

### Strengths and limitations

Our study has a number of strengths such as its big sample size, good response rate, community-based nature, and embedding in routine practice. A limitation may be the use of the CBCL, a parent-reported questionnaire, as criterion for the validation of other parent-reported short questionnaires. This use of the same informant could have increased indices for validity. Clinical assessments like psychiatric interviews may provide additional information. Because of their complexity and high costs, they were not used as criteria in this study. However, we were able to use treatment status as a criterion. Another limitation may be that the questionnaires to be assessed have a focus that slightly differs from the CBCL criterion. For example, the ASQ:SE encompasses a broader range of social and emotional behaviours than the CBCL. This could decrease agreement. As this is likely to affect all questionnaires to some degree, it will probably not affect our comparisons. We found somewhat lower validity indices when using treatment status as a criterion, although they were all in the same direction as the validity indices for an elevated CBCL score.

### Implications

Which of three short questionnaires (SDQ, ASQ:SE, KIPPPI) was most suitable for the identification of psychosocial problem among pre-school children? Regarding validity, only the SDQ discriminated sufficiently between 3–4 years old children with and without problems. Regarding use in community paediatric practice, the data were collected during routine practice, which supports the potential generalization of our results to this routine practice. Our findings showed that the SDQ adds more information to the identification in community paediatric practice than the KIPPPI and ASQ:SE. In sum, the SDQ can best be applied in community paediatric practice for the identification of psychosocial problems among pre-school children.

We found poorer psychometric properties of the Dutch ASQ:SE than of the US ASQ:SE. This may be caused by cultural differences or by differences in the interpretation of the items due to the translation of the ASQ:SE to Dutch. We recommend to replicate the validation of the ASQ:SE in other countries to investigate the cause of these differences.

Our findings imply that the use of short questionnaires, in particular the SDQ can improve the identification of psychosocial problems in community paediatric practice. Training in the use of the SDQ is needed to support the implementation of this questionnaire. As a next step after this identification, use of longer questionnaires and consultation of mental health specialists may help to target care to those most in need.

## Conclusions

Our comparison of the SDQ, KIPPPI and ASQ:SE showed that only the SDQ 3–4 had satisfactory psychometric properties and added value for community paediatric services. The SDQ can be considered as a useful aid for the early detection of psychosocial problems. Furthermore, the SDQ can provide effective support for community paediatric services in the identification of psychosocial problems among pre-school children. This instrument can therefore be validly applied in community paediatric practice.

## Abbreviations

PCH: Preventive child healthcare
CHP: Child healthcare professional
CBCL: Child behaviour checklist
SDQ: Strengths and difficulties questionnaire
PF: Parent form
KIPPPI: Short instrument for the psychological and pedagogical inventory’
ASQ:SE: Ages and Stages questionnaires: Social-Emotional
TDS: Total difficulties score
TPS: Total problems score
CFA: Confirmatory factor analysis
PCFI: Parsimony Comparative Fit Index
RMSEA: Root Mean Square Error of Approximation
OR: Odds ratio
CI: Confidence interval
AUC: Area under curve
